# New System for Tracking a Device for Diagnosing Scalp Ski

**DOI:** 10.3390/s140406516

**Published:** 2014-04-09

**Authors:** Hyung Gil Hong, Gi Pyo Nam, Hyeon Chang Lee, Kang Ryoung Park, Sung Min Kim

**Affiliations:** 1 Division of Electronics and Electrical Engineering, Dongguk University, 26 Pil-dong 3-ga, Jung-gu, Seoul 100-715, Korea; E-Mails: syutohell@nate.com (H.G.H.); oscar1201@dgu.edu (G.P.N.); leehc@dgu.edu (H.C.L.); 2 Department of Medical Bio Engineering, Dongguk University, 26 Pil-dong 3-ga, Jung-gu, Seoul 100-715, Korea; E-Mail: smkim@dongguk.edu

**Keywords:** scalp skin examinations, tracking the diagnostic device, color marker

## Abstract

In scalp skin examinations, it is difficult to find a previously treated region on a patient's scalp through images captured by a camera attached to a diagnostic device because the zoom lens on camera has a small field of view. Thus, doctors manually record the region on a chart or manually mark the region. However, this process is slow and inconveniences the patient. Thus, we propose a new system for tracking the diagnostic device for the scalp skin of patients. Our research is novel in four ways. First, our proposed system consists of two cameras to capture the face and the diagnostic device. Second, the user can easily set the position of camera to capture the diagnostic device by manually moving a frame to which the camera is attached. Third, the position of patient's nostrils and corners of the eyes are detected to align the position of his/her head more accurately with the recorded position from previous sessions. Fourth, the position of the diagnostic device is continuously tracked during the examination through images that help detect the position of the color marker attached to the device. Experimental results show that our system has a higher performance than conventional method.

## 1. Introduction

**Figure 1.**
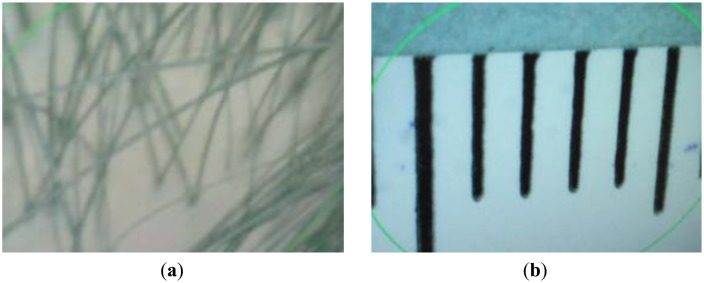
Sample images captured by a commercial device to diagnose a patient's skin. (**a**) Captured image including hair and scalp; (**b**) Image showing the zooming factor of the camera.

Devices equipped with cameras with high-magnification zoom lenses have been widely used to diagnose the treatment region on a patient's scalp [1–3]. Since a detailed view of the skin is needed, a high-magnification zoom lens is required to photograph it. Consequently, the field of view (FOV) of the camera is very small, as shown in Figure 1, where the vertical (black) lines show the gradations (markings) on a ruler. The green lines of Figure 1 are indication lines formed by the commercial device used in order to highlight the region of interest in diagnosing a patient's skin.

As shown in Figure 1b, a length of approximately 7 mm corresponds to that of 640 pixels in a 640 × 480 pixel image. Thus, a displacement of only 1 mm equals a shift of approximately 91 pixels in the image. As the field of view of the camera changes even with small movements, it is very difficult for doctors to locate again a previously treated region using the image captured by the camera. Thus, doctors manually record the regions in a chart, or manually mark it (e.g., by drawing a tattoo) to be able to relocate it for subsequent examination and diagnoses. However, these methods are slow and inconvenience the patient. To overcome these problems, we propose a new system to track the position of the device used to examine the skin on a patient's scalp.

Numerous studies have investigated methods to track medical devices [4–16]. These methods can be classified into two kinds: camera vision-based and sensor-based. Camera vision-based methods can be further classified into infrared light (IR) camera-based and visible camera-based methods [4–9]. Beller *et al.* proposed a method for tracking surgical instruments used during liver resection through an IR-based navigation system [4]. Fischer *et al.* proposed a medical augmented reality (AR) system that tracks surgical tools based on two IR cameras and IR reflective marker spheres [5]. Wang *et al.* used four IR cameras with IR lights and IR reflective marker spheres on a medical device to track the position of the device in three-dimensions (3D) [7]. In another study, they proposed a 3D tracking method for surgical instruments by using IR stereo cameras with IR illuminators and IR reflective marker spheres [8]. Duan *et al.* proposed a method for 3D tracking and positioning of surgical instruments using three visible-light cameras and markers for virtual surgery simulation [6]. Jianxi *et al.* proposed a technique for locating the 3D position of surgical instruments using binocular stereo cameras [9]. All of these studies have proposed methods to track and obtain the position of a medical device with reference to a fixed object. Furthermore, many of these methods involved the use of two or more cameras to obtain 3D position, which increases the cost of the system and requires camera calibration.

Sensor-based methods have also been extensively studied [10–16]. Yamaguchi *et al.* proposed a method for evaluating the laparoscopic suturing skills of experienced and novice surgeons by tracking a medical device with an electromagnetic motion tracker system [10]. Yamashita *et al.* proposed a real-time 3D model-based navigation system for endoscopic paranasal sinus surgery using an endoscope with an electromagnetic motion tracker sensor [11]. In another study, they proposed a system to track the 3D position of a laparoscopic instrument using an electromagnetic motion tracker for skills assessment and training [12]. Researchers have also studied clinical applications of tracking medical devices in 3D ultrasound-based systems [13–16]. All of these sensor-based methods require an additional device for tracking, which increases costs.

We propose a new, convenient, and cost-effective system for tracking devices used to diagnose the skin on a patient's scalp. The proposed system comprises two inexpensive web cameras to capture images of the face and a diagnostic device, along with equipment to immobilize the patient's chin and forehead. A user can easily position the camera to capture images through the diagnostic device by manually moving a frame to which the camera is attached. The position of the equipment used to immobilize the patient's chin and forehead can also be manually adjusted to the patient's height and the shape of his/her face. The system detects facial features, such as the nostrils and the corners of the eyes, to accurately align the patient's head according to its previously recorded position. The position of the diagnostic device is successively tracked through captured images by detecting the position of a color marker attached to the device. Table 1 summarizes the comparison between our proposed method and previous methods for tracking medical devices.

The rest of this paper is organized as follows: the proposed system and its method of implementation are described in Section 2, the experimental results are presented in Section 3, and the conclusions are drawn in Section 4.

## 2. Proposed System

### 2.1. Overview of Proposed System

**Figure 2.**
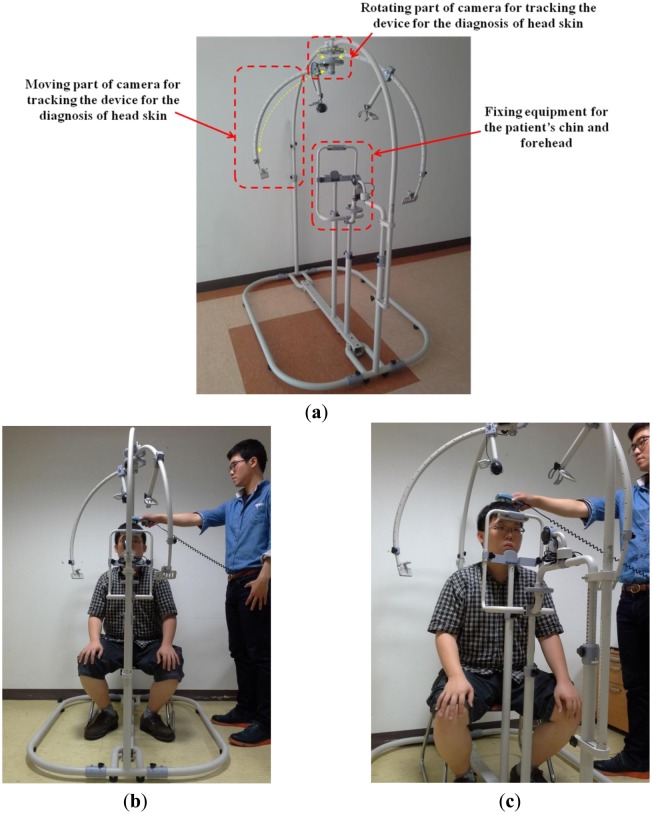
Proposed system for tracking the device to diagnose scalp skin. (**a**) Explanations of each part of proposed system; (**b**) Example of use of the proposed system (frontal view); (**c**) Example of use of the proposed system (another view).

**Figure 3.**
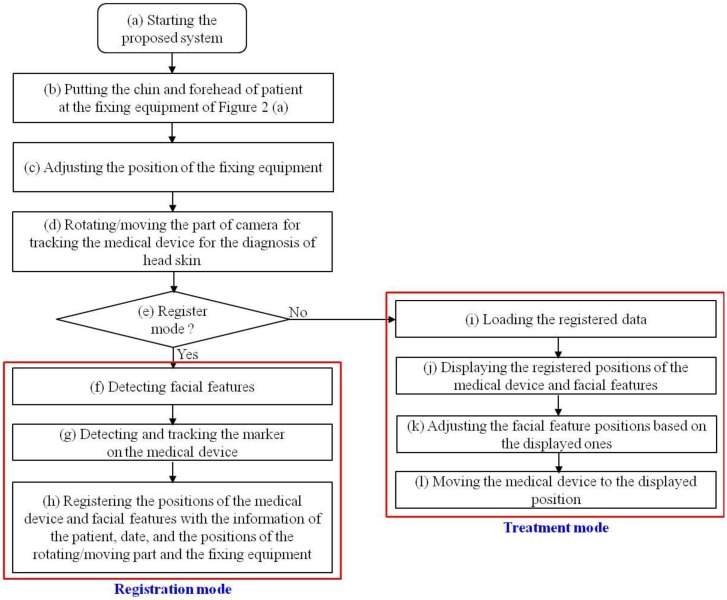
Overall procedure of the proposed method.

Figure 2a shows our proposed system for tracking medical devices used in diagnosing scalp skin. The system includes equipment to immobilize the patient's chin and forehead and the parts to move and rotate the camera to track the medical device. As shown in Figure 2b,c, a patient places his/her chin and forehead on the fixation equipment and the user rotates/moves the camera to capture the image of the medical device (see details in Section 2.2). The overall procedure is shown in Figure 3.

**Figure 4.**
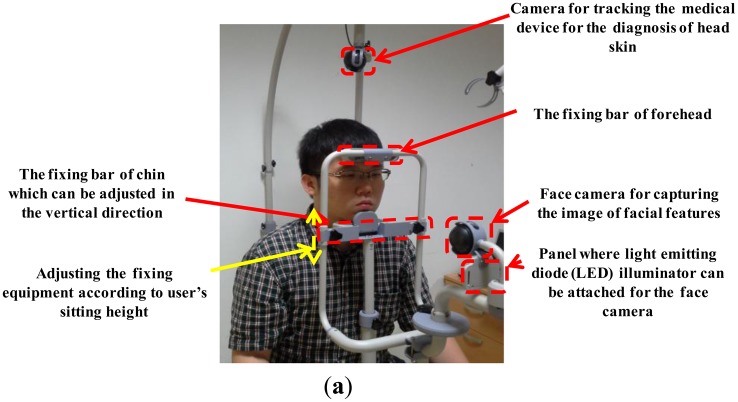

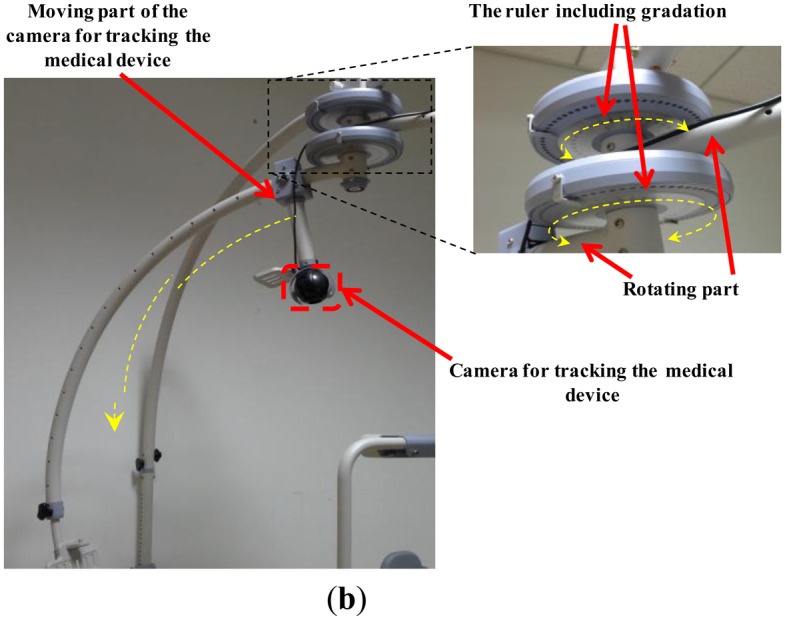
Proposed system. (**a**) Equipment to immobilize the chin and forehead of a patient; (**b**) Rotating/moving part of camera for tracking the medical device.

The patient places his/her chin and forehead on the fixation equipment, as shown in Figure 2b,c. Since sitting height varies among people, our system allows the user (doctor) to adjust the position of the fixation equipment. The user then manually rotates and moves the camera to track the medical device. When the system is in registration mode, the facial features of the patient can be detected either manually or automatically. The center and direction of the marker attached to the medical device can then be detected either manually or automatically. All the patient's facial features (the inner corners of the eyes and both nostrils) and the positions with directions of the medical device are registered along with the patient's data, the date, and the positions of the rotating/moving part of the camera and the fixation equipment of Figure 4. When in treatment mode, our system loads the patient's registered data, and displays the patient's facial features and the registered positions with directions of the medical device on a monitor. The user can then accurately adjust the position of the facial features based on the ones displayed. Following this, the user moves the medical device to the previously registered display position.

### 2.2. Detailed Explanation of Proposed System

The system can be disassembled, which makes it easy to move. As shown in Figure 4a, the patient places his/her chin and forehead on the fixation bars. The fixation bar for the chin can be vertically adjusted to accommodate different facial sizes. Moreover, by vertically adjusting both the chin and the forehead fixation bars, the system is able to accommodate different sitting heights. As Figure 4a shows, the web camera captures 24-bit color images (1,600 × 1,200 pixels) of the face and facial features are detected in the image (see Section 2.3). To obtain the image of the face even in low illumination, additional light-emitting diode (LED) illuminators can be attached below the camera that captures the patient's facial features. Even though the user places his/her chin and forehead on the fixing bar, the position of the head can vary because it is not tied to the fixing bar and because the chin and forehead shapes vary from person to person. Thus, an additional camera for tracking facial features is required in our system. By adjusting the position of the patient's head based on his/her facial features, the position of the optical device used in diagnosing scalp skin can be detected more accurately.

**Figure 5.**
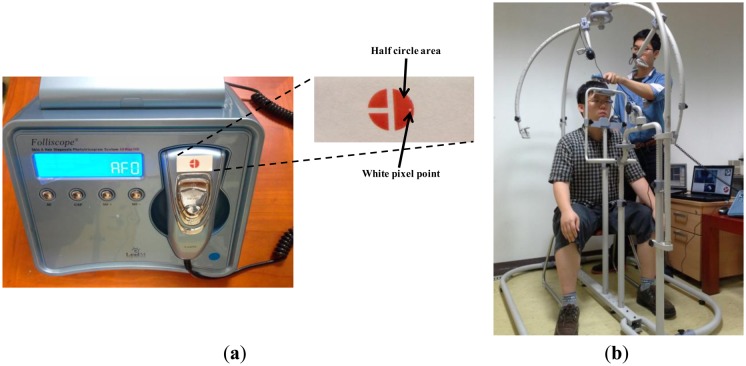
Commercial diagnostic device for scalp skin diagnosis and its use. (**a**) Commercial diagnostic device with attached color marker; (**b**) Example of using the device.

Figure 4b shows the web camera used to track the medical device. It also captures a 24-bit color image (1,600 × 1,200 pixels). The camera can be easily rotated (from 0 to 360 degrees) and moved to locate the medical device. Since this part is graduated, the positions can also be manually recorded. As explained in Section 2.1, based on the recorded positions, the doctor can easily set the positions for subsequent treatment sessions. The device is attached to the patient's head which is not planar shape. If the camera is not positioned above the device, it can capture the image of device at a slant. Consequently, the shape of the color marker on the device, shown in Figure 5a, can be distorted in the captured image, which can degrade the accuracy with which the position and direction of the device is detected. In addition, the light reflection on the surface of marker can usually occur by the environmental light, which can reduce the detection accuracy of the marker. Thus, it is necessary to move the moving part with the camera and capture the marker image in different direction so as to prevent these cases of the distortion of marker shape and light reflection on the surface of the marker.

### 2.3. Tracking the Position of the Medical Device and Locating Facial Feature Positions

Figure 5 shows a commercial diagnostic device, Folliscope [1], used for scalp skin diagnosis. As shown in Figure 5a, we create a color marker and attach it to the device to detect the center and direction of the marker. We can also attach a near-infrared (NIR) LED to the device instead of using a color marker. However, this requires an additional power supply module, such as a battery or a power line, which increases the weight of the device and thus reduces user convenience. Hence, we use a color marker on the device, which is tracked by a conventional web camera.

**Figure 6.**
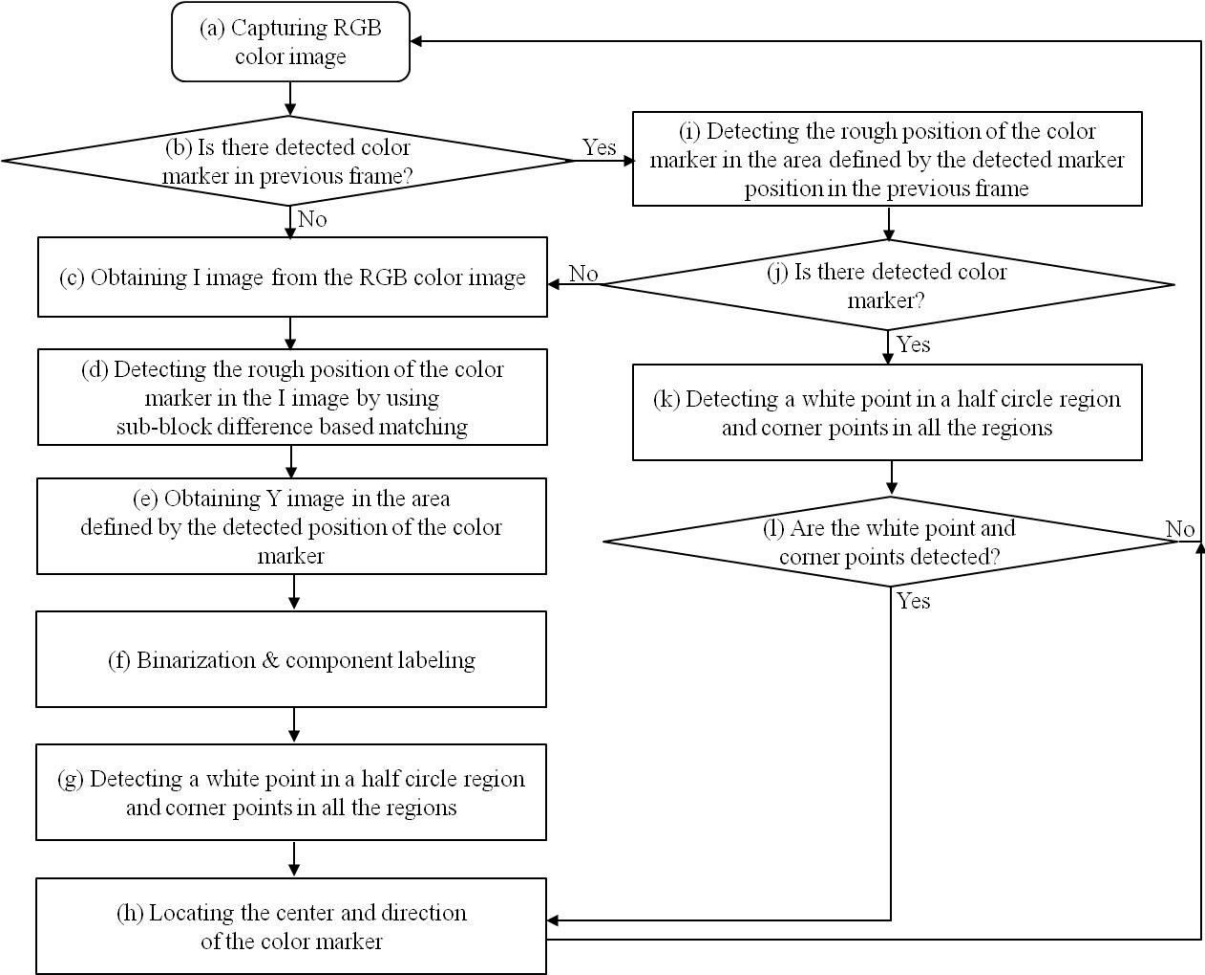
Flowchart of detection process of the center and direction of color marker.

**Figure 7.**
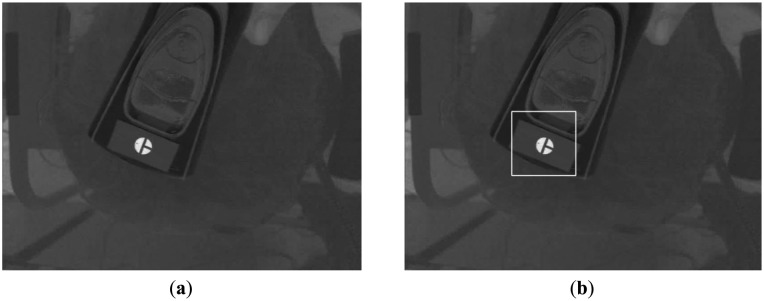
Color marker in the I image and its detection. (**a**) Red-colored marker in the I image. (**b**) Detection of marker by sub-block difference-based matching.

As shown in Figure 5b, a doctor places the device on the patient's head and observes the captured image on the monitor. As Figure 5b shows, an image of the patient's scalp as well as the diagnostic device is captured, and the center and direction of the color marker are detected based on the procedure depicted in Figure 6. The captured RGB image is transformed into an in-phase (I) image in the YIQ color space. We obtain only the I image (without Y and Q images) from the RGB image to increase processing speed. In general, the RGB color model includes information about the color and the brightness, and is affected by change of illumination. Since the I image is less affected by variation in illumination than the RGB image, and since the red color of the marker (Figure 5a) appears as bright pixels in the I image (Figure 7a), it can be easily detected.

Using the I image shown in Figure 7a, we detect the marker using sub-block difference-based matching. This method is based on our past research [17].

**Figure 8.**
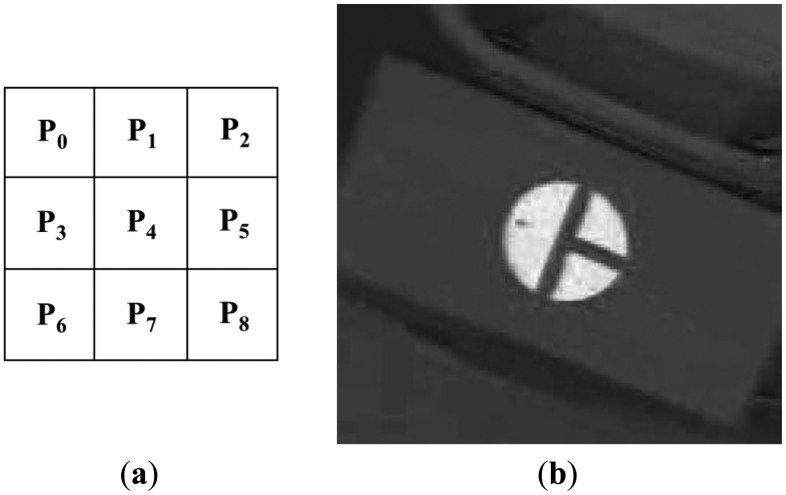
Sub-block difference-based matching using 3 × 3 sub-block mask. (**a**) 3 × 3 Sub-block mask; (**b**) Detected marker position using 3 × 3 sub-block mask.

Figure 8a shows the 3 × 3 sub-block mask. P_i_ (*i* = 0–8) represents the average gray value of the P_i_ sub-block. As shown in Figure 8b, the red color of the marker appears as bright pixels in the I image. Thus, the calculated sub-block difference-based matching score (SDMS) by Equation (1) [17] is maximized at the position where the 3 × 3 sub-block fits the marker, as shown in Figure 8b. Since the size of the marker in the image will vary according to the Z distance between the camera and the medical device, the mask shown in Figure 8a is scanned at various sizes over the entire area of the I image, and the position that maximizes the SDMS score is determined to be the final marker position. In order to speed up the processing time to calculate P_0_–P_8_, an integral imaging scheme is used [17]:

(1) $$\text{SDMS}=\sum_{i=0}^{3}\left\{|{\text{P}}_{4}-{\text{P}}_{\left(2\times i+1\right)}\right|\}$$

**Figure 9.**
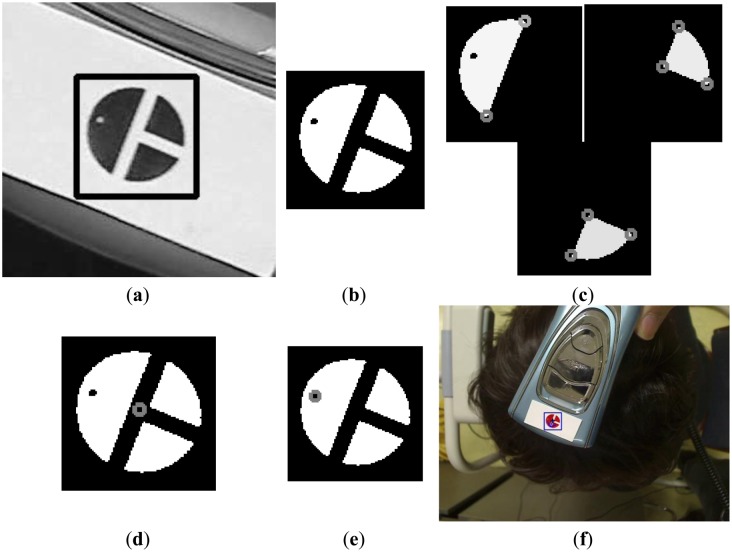
Detection of center and direction of the marker. (**a**) Obtained Y image based on marker position detected by sub-block difference-based matching; (**b**) Result of binarization and component labeling of the Y image; (**c**) Detection of the eight corner positions; (**d**) Detection of center of marker; (**e**) Detection of white point in the semi-circular area; (**f**) Results of detecting the direction and center of the marker.

Figure 7b shows the detection of the marker by sub-block difference-based matching. Based on the detected area of the marker, the defined area of the Y image in the YIQ color space is obtained, as shown in Figure 9a. Here, we obtain only the Y image (not the Q image) from the RGB image in the defined area in order to increase processing speed. We perform the binarization and component labeling with the Y image to obtain the three regions shown in Figure 9b. With all three regions, we detect the corner points using a Harris corner detector [18], as shown in Figure 9c. Using the eight corner points, we can calculate the center of the marker, as shown in Figure 9d. Figure 9b shows the detection of the semi-circular region (Figure 5a) with the largest size; and inside the semi-circular area, the white pixel point (Figure 5a) is detected by binarization and component labeling in the area defined by the line, as shown in Figure 9e. The line is determined by the midpoints of the two lower corner points (the central image of Figure 9c) and the two upper corner points (the right-hand image of Figure 9c). Using the center (Figure 9d) and the white pixel point (Figure 9e) of the marker, we can calculate the direction of the diagnostic device (the arrow in Figure 9f). In addition, the center of the marker is determined to be the position of the diagnostic device (the cross-mark in Figure 9f).

As shown in step (b) of the flowchart in Figure 6, if the marker position is detected in the preceding frame, the procedures shown in Figure 7b and Figure 9 a–e are performed in the region defined using the detected position in the previous frame, as shown in steps (i)–(l) in the flowchart in Figure 6.

**Figure 10.**
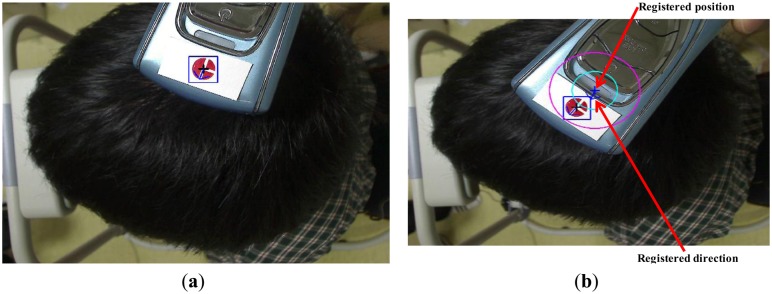

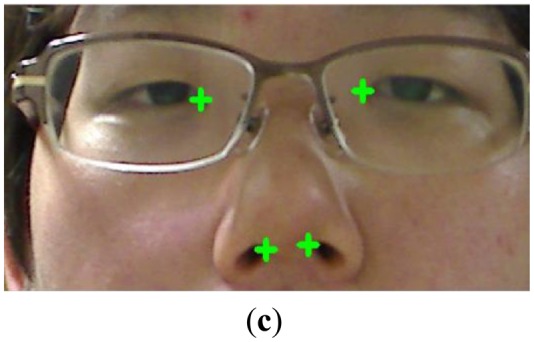
Detected center and direction of color marker and facial feature positions. (**a**) Detected center and direction of marker (registration mode in Figure 3); (**b**) Moving the diagnostic device to the registered position and in the direction of the marker (treatment mode in Figure 3); (**c**) Manually detected facial feature points.

Figure 10a shows the results of the detection of the center and the direction of the marker. When the system is in treatment mode, the registered center (cross-mark in Figure 10b) and the direction (arrow in Figure 10b) of the marker are displayed, as shown in Figure 10b, and the user attempts to fit the center and the direction of the marker into the ones displayed. By displaying two additional circles (the light-blue and violet circles in Figure 10b) based on the accurately registered center position and the direction of the marker, our system helps the user quickly move the device to the correct position. The violet circle shows the rough region of the registered position of the center of the marker, while the blue one represents a more accurate region of the same. By displaying these two circles, the user (doctor) can easily fit the position of the center of the marker on the device to the accurately registered position (the dark blue cross-mark within the blue circle of Figure 10b).

Since the small area of the scalp skin is magnified in the image captured by the camera, as shown in Figure 1, the change of direction of the marker can make it difficult for the user to find the correct position of the device. Thus, our system detects the position and direction of the marker as shown in Figure 9f.

Although the fixation bars are designed to keep the patient's head and chin in place during examination, as shown in Figure 3a, the position of the patient's chin and forehead are bound to be slightly different from those in each of the previous registration modes. Consequently, although the doctor makes the marker fit the registered center and direction, they can be different from previously registered positions. In order to solve this problem, our system also registers the position of the facial features of the patient in registration mode. As shown in Figure 10c, the inner corners of the eyes and both nostrils are detected either manually or automatically in registration mode, and are displayed in treatment mode. The doctor then moves the patient to fit the position of his/her facial features with the accurately displayed positions.

During automatic detection of the position of the facial feature in registration mode, we use sub-block difference-based matching to detect the outer corners of the eyes. We use binarization to detect both nostrils and to obtain the geometric center of each nostril. The position of the eyeballs changes according to the direction of the patient's gaze, and can cause a variation in the position of the patient's head if used as a reference point (adjusting the user's head on the fixing bar of our device based on the positions of the eye balls). Thus, we use the corners of the eyes to adjust the position of the patient's head in our system, as shown in Figure 10c.

## 3. Experimental Results

Our experiments involved 22 participants. The average age of the participants was 27.4 (a standard deviation of 1.9). They took part in the experiments voluntarily. Twenty people (testers) acted as doctors and attempted to fit the center of the marker on the diagnostic device with its registered position, and the other two (subjects) acted as patients. Each tester made 16 attempts using both our proposed system as well as conventional methods for device detection. In particular, after dividing the area occupied by the patients' head into four sub-regions, four target positions per sub-region were randomly selected for diagnosis. Since a user's head is not planar shape and we placed the device on the surface of the head, the image of the marker on top of the head is different from those in the four sub-regions. Thus, we performed the experiments in the four sub-regions. For the conventional method, the testers first recorded the position of the diagnostic device on the patient's head on a medical chart, and then attempted to find the position by referring to the chart.

In the first experiment, we measured the detection error for the center and the direction of the marker by using our proposed marker detection algorithm shown in Figure 6. To measure the performance for various scanning directions of the diagnostic device, we obtained image sequences as follows. First, the tester moved the device in a zigzag pattern in the horizontal direction. Second, the tester moved the device in a zigzag pattern in the vertical direction. In each scanning direction, we obtained three image sequences under different illumination conditions: fluorescent lighting from the left, middle, and right above the head. Each 10 s sequence consisted of 150 frames, and we obtained the sequences in an office environment mimicking that of a hospital. The detection error of the marker center is calculated by the Euclidean distance between the manually detected center and the center calculated by our proposed method. To measure the detection error of the marker direction (angle), we manually detected the center and the white pixel point of the semi-circular area of the marker (Figure 5a). From this, we calculated the ground-truth (angle) in the counter-clockwise direction by fixing the three o'clock direction as 0 degrees. The difference between the angle calculated manually and that calculated by our proposed method was considered the detection error in marker direction. As shown in Table 2, the average error in the detection of the center of the marker is approximately 2.12 pixels, and that in the detection of marker direction is 2.25 degrees. These results confirm that our method can accurately detect the center and direction of the marker.

**Figure 11.**
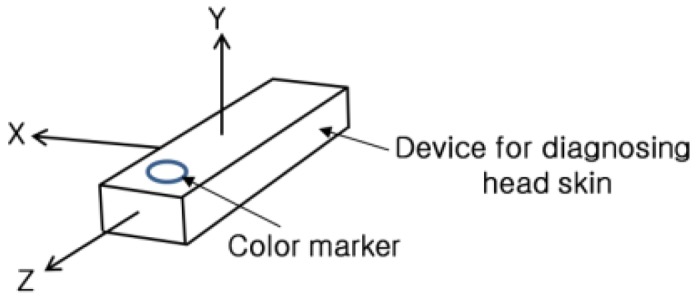
Axes for movement of device for experiments.

In our second experiment, we tested the translation, rotation and scaling of the position of device, as well as its affine invariance. Based on the Figure 11, we obtained 625 images for translation along the *x*-axis and the *y*-axis (5 users × 5 trials × 5 steps of translation (scaling) based on the *y*-axis × 5 steps of translation based on the *x*-axis), and another 625 images for translation along the *y*-axis and the *z*-axis (5 users × 5 trials × 5 steps of translation (scaling) based on the *y*-axis × 5 steps of translation based on the *z*-axis). We acquired an additional 750 images (5 users × 5 trials × 5 steps of translation (scaling) based on the *y*-axis × 6 steps of rotation based on the y-axis). Hence, a total of 2,000 images were obtained for the experiment.

The experimental results are shown in Tables 3, 4 and 5. The numbers of translations based on the *y*-axis represented the distance between the marker on the device and the camera used to track the marker. Since the size of marker changes in the image according to the distance between the device and the camera, we can refer to the translation based on the *y*-axis as the scaling based on it. Because we consider the movement of the device based on affine invariance, the data from the rotation based on the *y*-axis were used for the experiment. Rotations based on the *x*- and *z*-axes of Figure 11 are not allowed because they produce distorted (affine variant) images of the marker. The results of the detection error in view of affine variance are presented in Table 2.

As shown in Tables 3, 4 and 5, the device detection accuracy of our method is not affected by the translation, rotation and scaling of the position of the device, and is similar to those in the case of affine variance of Table 2.

**Figure 12.**
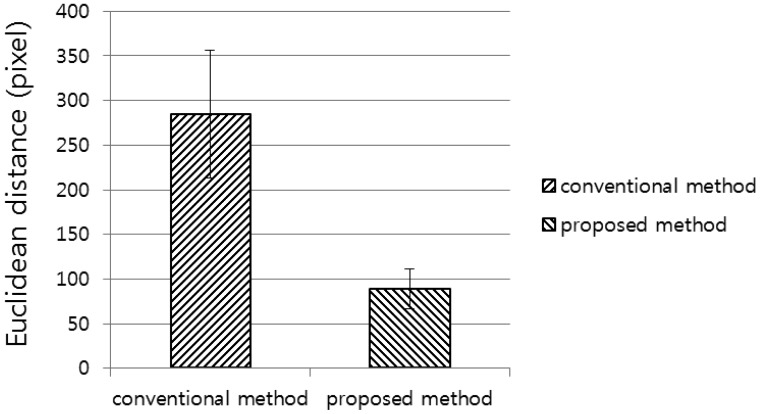
Comparisons of Euclidean distance between the conventional and proposed methods.

In the third experiment, we compared the detection error of the proposed method with that of the conventional method. Here, the conventional method means that the testers first recorded the position of the diagnostic device on the subject's head on a medical chart, and then attempted to find the position by referring to the chart. The detection error is calculated in terms of the Euclidean distance between the registered center position of the marker and the detected position. The experimental results showed that the detection error of our proposed method was smaller than that of the conventional method (Figure 12). A two-tailed *t*-test was performed based on the standard deviations and the means of each group [19] to analyze the statistical significance between the two datasets. The calculated *p*-value of 4.04 × 10^−21^ clearly shows that the detection error of the proposed method is significantly smaller than that of the conventional method, with a confidence level of 99% (0.01) [19]. We used as null-hypothesis the claim that there is no difference between the detection error of the proposed method and that of the conventional method in Figure 12. According to the principle of the *t*-test [19], if the *p*-value is less than the confidence level, the null-hypothesis is rejected, which represents that there exists a difference between the detection error of the proposed method and that of the conventional method.

**Figure 13.**
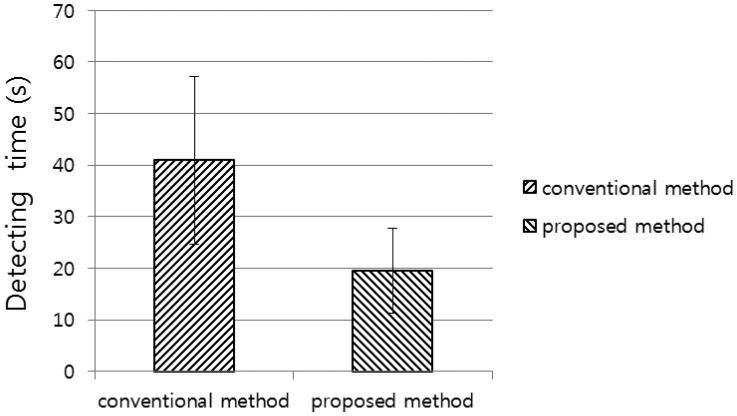
Comparison of detection time between the conventional and proposed methods.

In the next experiment, we compared the detection time of the proposed method with that of the conventional method. The detection time is the time taken by a tester to successfully locate the registered center position of the marker. The experimental results showed that the detection time of the proposed method was less than that of the conventional method (Figure 13). A *p*-value of 5.39 × 10^−10^ proves that the detection time of the proposed method is significantly less than that of the conventional method, with a confidence level of 99% (0.01) [19]. We used as null-hypothesis that claim that there is no difference between the detection time of the proposed method and that of the conventional method in Figure 13.

**Figure 14.**
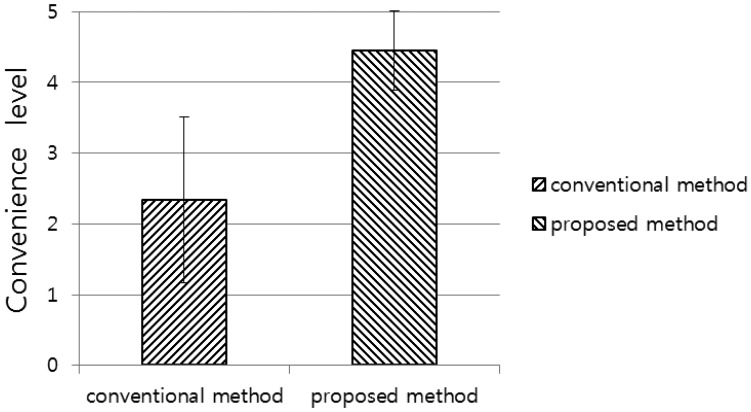
Comparison of convenience level between the conventional and proposed methods.

In our final experiment, we performed a subjective test. We evaluated the convenience of using the proposed and conventional systems with the 20 testers. Convenience was evaluated based on a five-point scale (1: very uncomfortable, 2: uncomfortable, 3: usual, 4: comfortable, 5: very comfortable). As shown in Figure 14, the convenience of using the proposed system is higher than that of using the conventional system. A *p*-value of 1.61 × 10^−14^ clearly indicates that the convenience level of the proposed system is significantly higher than that of the conventional system, with a confidence level of 99% (0.01) [19]. We used as null-hypothesis the claim that there is no difference between the convenience level of the proposed method and that of the conventional method in Figure 14.

As an alternative, a linkage-type robot with an encoder can be used in our system to make it even more convenient for the user. However, it would increase the weight, size, and cost of the system. Our study is aimed at making a lighter, smaller, and cheaper system, which can easily be used and moved by a doctor. From the outset, we consulted a medical doctor and referred to his recommendations in order to capture and address with our system the needs of clinical practitioners. Hence, based on these information, we designed our system without a linkage-type robot with an encoder.

## 4. Conclusions

We proposed a new system to track the device used to diagnose scalp skin. This system comprises two cameras for capturing images of the face and a diagnostic device to immobilize the chin and forehead of the patient. Facial features, such as the nostrils and the corners of the eyes, are detected to align a patient's head accurately with its position as recorded in previous sessions. Our system continuously tracks the position of the diagnostic device during the examination using images to identify the position of a color marker attached to the diagnostic device. Experimental results show that the proposed system has a higher accuracy, can detect the treatment region faster, and is more convenient for the user than the conventional method. Reflecting light on the surface of the marker can reduce its detection accuracy. In our system, this problem is avoided by moving the moving part of camera with it. Further, the quick movement of the device can make it difficult for the user to find its correct position. In future work, we plan to research methods to increase the detection accuracy and speed of our system by using additional sensors or multiple camera systems.

## Figures and Tables

**Table 1.** Comparison between proposed method and previous methods for tracking medical devices.

**Category**			**Method**	**Strength**	**Weakness**
Camera vision-based method	IR-camera-based method		Two or more cameras are used to track the 3D position of IR reflective marker spheres on surgical instruments [5,7,8]	Accurate 3D position of surgical instrument can be obtained quickly	The position of the object to be measured by the medical device is assumed to be fixed The system is expensive Camera calibration is required
	Visible light camera-based method	Multiple camera-based method	Two or more cameras are used to track the 3D positions of the medical device [6,9]	Additional IR illuminators and IR reflective markers are not required	
		Single camera-based method (Proposed method)	One camera is used to track the 2D position of the medical diagnostic device	Additional IR illuminators, IR reflective markers, and camera calibration are not required The position of the object to be measured by the medical device can also be tracked by another camera	2D position of medical device can be obtained instead of 3D position
Sensor-based method	Electromagnetic sensor-based method		3D position of surgical instrument is tracked by the attached electromagnetic motion tracker [10–12]	The position of the medical device can be tracked and is not affected when occluded by the object or when inside the object	The system is expensive
	Ultrasound-based method		Ultrasound-based tracking of medical device [13–16]		

**Table 2.** Detection error results for distance and angle of marker center pixel.

**Zigzag Scanning Direction**	**Light**	**Error of Marker Center (Unit: Pixels)**	**Error of Direction (Angle) of Marker (Unit: Degrees)**
Horizontal direction	Mid	1.78	0.90
	Right	1.63	2.83
	Left	2.36	2.39

Vertical direction	Mid	2.24	2.36
	Right	1.88	2.65
	Left	2.81	2.39

Average		2.12	2.25

**Table 3.** Detection error results in case of the translation of device based on *x*- and *y*-axes, respectively.

			**Translation Based on *x* axis**					**Average**

			−3 cm	−1.5 cm	0 cm	1.5 cm	3 cm	

Translation (scaling) based on *y* axis	10.7 cm	Error of marker center (unit: pixels)	1.98	2.45	2.34	2.77	2.13	2.33
	11.7 cm		1.84	1.43	2.21	2.41	2.68	2.11
	12.7 cm		2.75	1.66	2.36	3.53	2.61	2.58
	13.7 cm		1.84	2.42	2.89	2.67	2.79	2.52
	14.7 cm		2.30	2.42	2.16	2.58	2.91	2.47

	Average		2.14	2.08	2.39	2.79	2.62	2.40

	10.7 cm	Error of direction (angle) of marker (unit: degrees)	0.90	1.36	1.58	1.38	1.49	1.34
	11.7 cm		1.08	1.07	1.30	1.33	3.98	1.75
	12.7 cm		1.07	0.84	0.78	2.92	1.21	1.36
	13.7 cm		0.94	1.05	1.37	0.98	2.12	1.29
	14.7 cm		1.49	1.00	2.34	1.42	1.52	1.55

	Average		1.10	1.06	1.47	1.61	2.06	1.46

**Table 4.** Detection error results in case of the translation of device based on *y*- and *z*-axes, respectively.

			**Translation Based on *z* axis**					**Average**

			−3 cm	−1.5 cm	0 cm	1.5 cm	3 cm	

Translation (scaling) based on *y* axis	10.7 cm	Error of marker center (unit: pixels)	2.30	3.12	1.81	2.26	2.53	2.40
	11.7 cm		2.99	1.73	2.55	1.47	2.22	2.19
	12.7 cm		2.31	2.50	2.05	2.05	2.50	2.28
	13.7 cm		2.49	2.60	2.29	2.45	2.17	2.40
	14.7 cm		1.71	2.35	2.08	2.52	2.99	2.33

	Average		2.36	2.46	2.16	2.15	2.48	2.32

	10.7 cm	Error of direction (angle) of marker (unit: degrees)	1.19	1.58	1.53	1.14	1.25	1.34
	11.7 cm		3.03	1.26	1.23	1.28	1.59	1.68
	12.7 cm		1.10	1.46	1.33	1.15	0.91	1.19
	13.7 cm		1.18	1.36	1.45	1.20	1.16	1.27
	14.7 cm		1.55	1.46	1.80	1.18	1.6	1.52

	Average		1.61	1.42	1.47	1.19	1.30	1.40

**Table 5.** Detection error results in case of the translation and rotation of device based on *y*-axis, respectively.

			**Rotation Based on *y* axis**						**Average**

			0	60	120	180	240	300	

Translation (scaling) based on *y* axis	10.7 cm	Error of marker center (unit: pixels)	2.21	2.89	4.38	4.19	1.73	4.81	3.37
	11.7 cm		2.86	2.24	2.73	2.56	1.15	2.23	2.30
	12.7 cm		2.17	2.20	1.77	2.41	1.90	2.34	2.13
	13.7 cm		2.79	1.89	1.77	2.45	1.83	1.47	2.03
	14.7 cm		2.82	3.04	2.88	2.14	1.90	2.08	2.48

	Average		2.57	2.45	2.71	2.75	1.70	2.59	2.46

	10.7 cm	Error of direction (angle) of marker (unit: degrees)	1.42	5.94	3.25	4.08	1.93	1.06	2.95
	11.7 cm		1.15	1.76	2.44	2.26	1.73	0.99	1.72
	12.7 cm		1.08	2.36	1.27	2.16	1.28	1.19	1.56
	13.7 cm		1.07	2.30	1.31	2.45	0.91	1.30	1.56
	14.7 cm		0.91	2.11	2.54	2.02	1.06	1.46	1.68

	Average		1.13	2.89	2.16	2.59	1.38	1.20	1.89
